# Gelsolin variant amyloidosis mimicking progressive bulbar palsy

**DOI:** 10.1002/mus.27714

**Published:** 2022-09-21

**Authors:** Jinseok Park, Young‐Eun Kim, Ki‐Wook Oh, Minyeop Nahm, Yu Jeong Kim, Mi Jung Kim, Seung Hyun Kim

**Affiliations:** ^1^ Department of Neurology College of Medicine, Hanyang University Seoul South Korea; ^2^ Cell Therapy Center Hanyang University Hospital Seoul South Korea; ^3^ Department of Laboratory Medicine College of Medicine, Hanyang University Seoul South Korea; ^4^ Dementia Research Group, Korea Brain Research Institute Daegu South Korea; ^5^ Department of Ophthalmology Hanyang University College of Medicine Seoul South Korea; ^6^ Department of Rehabilitation Medicine Hanyang University College of Medicine Seoul South Korea

**Abbreviations**
EMG: electromyography
GSN: gelsolin
MND: motor neuron disease
PBP: progressive bulbar palsy

Hereditary gelsolin amyloidosis, a rare autosomal dominant hereditary disease, is caused by pathogenic variants in the gelsolin (*GSN*) gene.^1^ It is characterized by slowly progressive cranial and peripheral nerve degeneration, corneal lattice dystrophy, and loss of skin turgor (cutis laxa).^1^ However, it may be misdiagnosed as slowly progressive motor neuron disease (MND) or progressive bulbar palsy (PBP) owing to symptomatic similarity.^2^ Here, we describe two patients with GSN amyloidosis with a long‐lasting undiagnosed MND‐mimicking presentation that was eventually confirmed by genetic testing.

Patient A had visited our MND clinic 2 y ago. She was a 58‐y‐old woman with a 6 y history of progressive facial diplegia, slurred speech, difficulty swallowing water, and tongue atrophy. Neurological examination revealed bilateral lower facial weakness, myokymia, tongue atrophy, and dysarthria. There were no sensory abnormalities, and reflexes were normal in all extremities. There was no family history of neuromuscular disease. She had dry eye syndrome, but no other past medical history. Blood tests for thyroid disease, autoimmune disease, vasculitis, paraneoplastic syndrome, paraproteinemia, and infectious diseases were normal or negative. Sensory and motor nerve conduction studies of the upper and lower extremities were normal. Positive sharp waves and fibrillation potentials were noted in the orbicularis oris and masseter muscles on needle electromyography (EMG). Brain magnetic resonance imaging was unremarkable. Genetic testing using the TruSight One Sequencing Panel (Illumina Inc., San Diego, CA), which contains 4811 genes related to MND, did not reveal a known pathogenic variant.

Patient B was a 60‐y‐old woman with a 4 y history of dysarthria, tongue atrophy, and facial twitching. Her family members had similar features without a diagnosis. Neurological examination revealed facial diplegia with ptosis, decreased skin turgor, twitching of facial muscles, tongue atrophy, and dysarthria with normoactive reflexes in all extremities (Figure 1). There were no pathologic upper motor neuron signs. No sensory or visual symptoms were reported. Motor and sensory nerve conduction studies of the right upper and lower extremities were normal. Needle EMG revealed fibrillation potentials, positive sharp waves, and large motor unit potentials with reduced recruitment in the masseter and tongue muscles. Her grandmother (I‐2), mother (II‐2), and elder brother (III‐1) showed similar symptoms (Figure 2B). We performed whole‐exome sequencing and detected a pathogenic variant, c.640G > T (p.D214Y), in the *GSN* gene of the patient and sibling III‐1. Sibling III‐3 was asymptomatic and had no pathological variants (Figure 2D).

**FIGURE 1:**
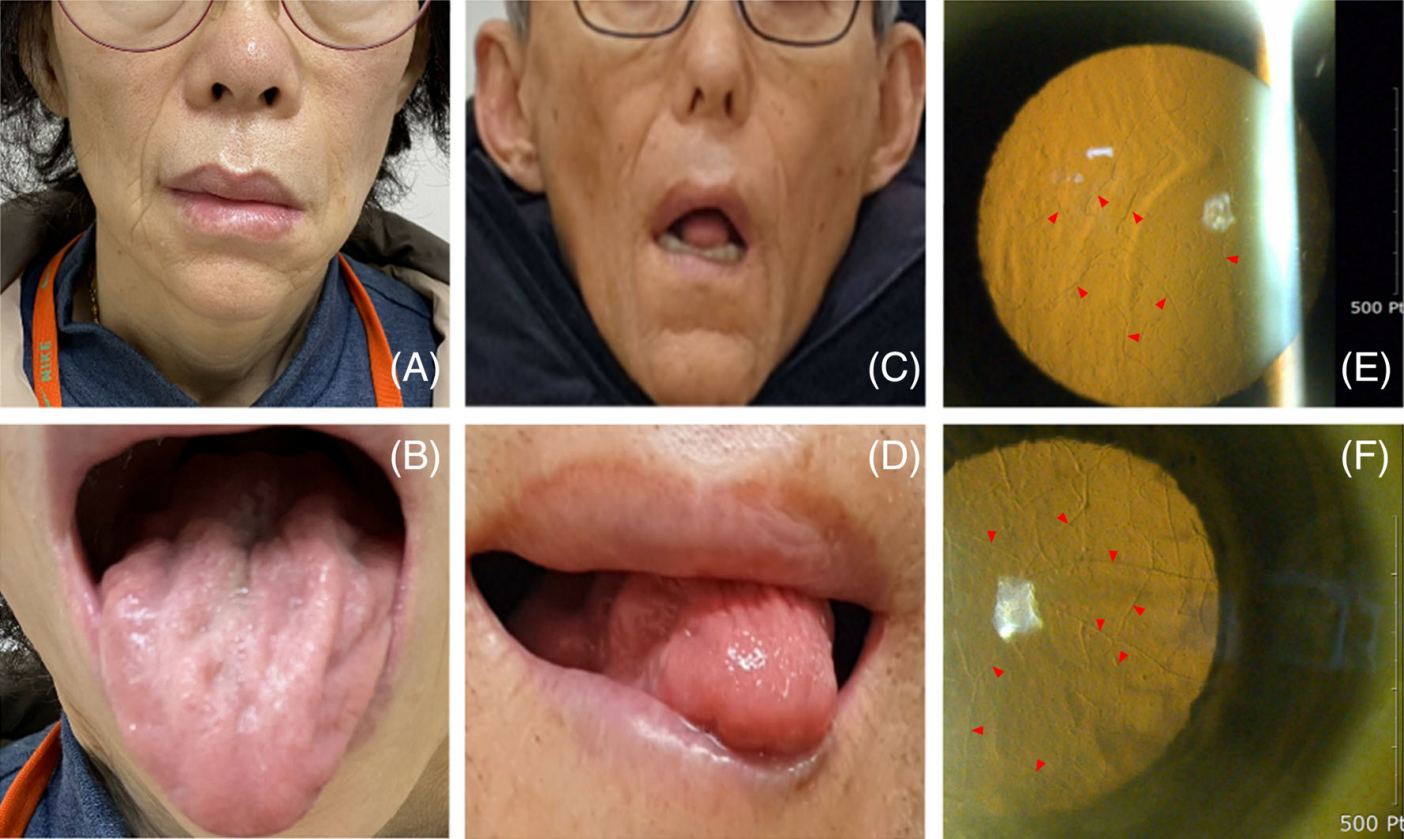
Patient B with left‐dominant facial diplegia (A) and an atrophied tongue (B). Her elder brother (III‐1) had a similar appearance (C, D). On ophthalmologic examination, small dots and branching filamentary lines with amyloid deposits (red wedges) can be seen in the corneal stroma of patients A (F) and B (E)

**FIGURE 2:**
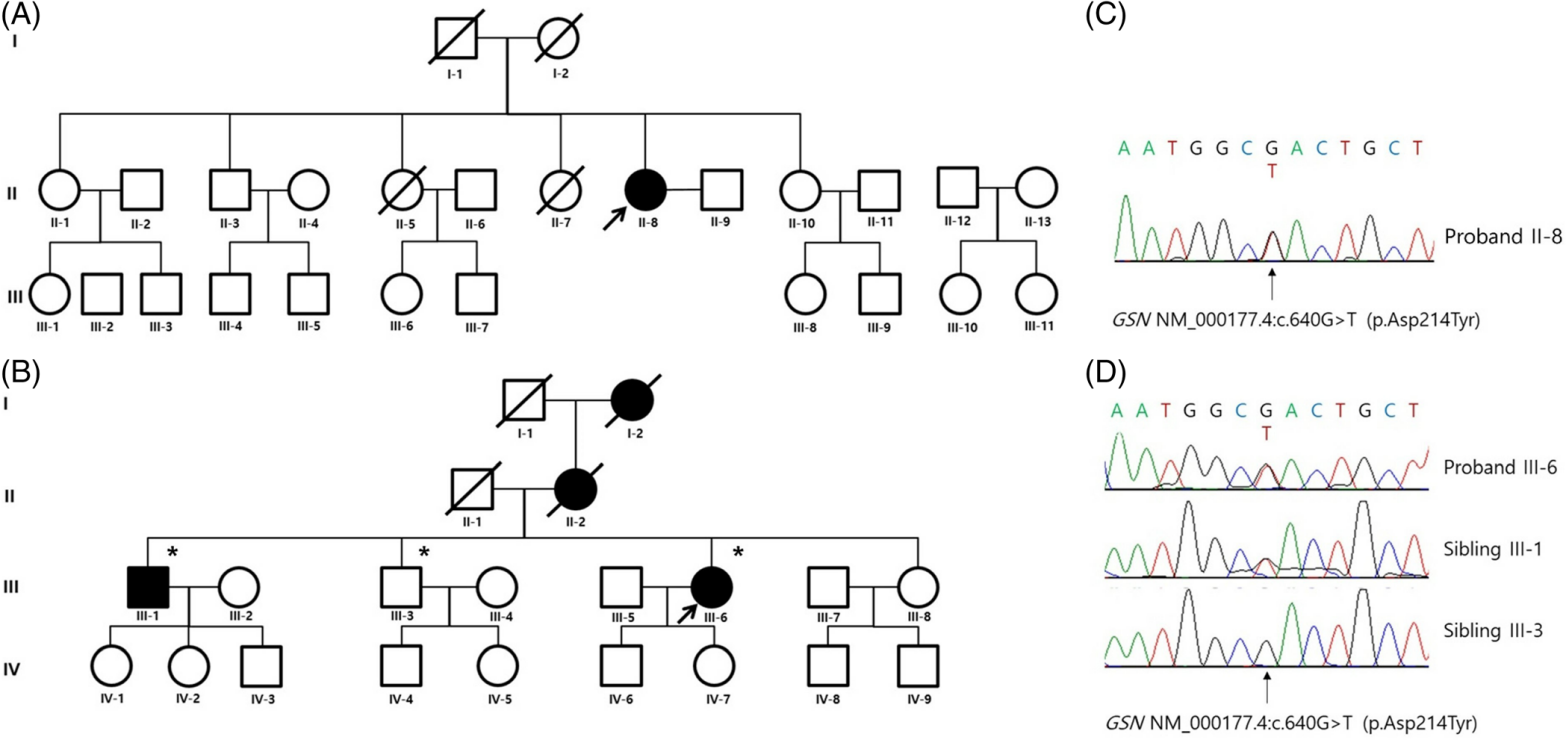
Pedigrees and sequence chromatograms of patients with gelsolin (GSN) amyloidosis. (A) Patient A (black arrow) was the only one with neurological symptoms compatible with GSN amyloidosis in her family. (B) *GSN* genetic testing was performed for patient B (black arrow) as a proband and her siblings (asterisks). In sequence chromatograms, pathogenic *GSN* variants were found in patient A (C) and patient B (III‐6) and her symptomatic sibling (III‐1) (D). (D) Asymptomatic sibling of patient B (III‐3) had no pathogenic *GSN* variant

After noting the genetic findings in patient B, we performed whole‐exome sequencing on patient A. The *GSN* variant (p.D214Y) was identified (Figure 2A and C). Subsequently, both patients were diagnosed with corneal lattice dystrophy by an ophthalmologist using slit‐lamp examination with the retro‐illumination technique (Figure 1E and F).

To date, several pathogenic *GSN* variants have been reported, including peripheral neuropathy, corneal lattice dystrophy (c.640G > A, c.640G > T, c.1631T > G), and chronic kidney disease (c.580G > A, c.633C > A). Recently reported rare phenotypes are seizures (c.100dupG), dermatomyositis‐like symptom (c.1375C > G), and cardiac involvement (c.1783G > A, c.1732G > C). Most of the reported cases of *GSN* variants are familial.^3^ In diagnosing hereditary gelsolin amyloidosis, positive family history and characteristic ophthalmic findings, such as amyloid deposition on the cornea, are important early diagnostic clues.^1^ However, as observed in patient A, it is difficult to diagnose even a known *GSN* variant if there is no family history. Corneal lattice dystrophy is another unique ophthalmic characteristic of hereditary GSN amyloidosis and is reported to be an initial manifestation of gelsolin amyloidosis. However, patients A and B were not diagnosed with corneal lattice dystrophy through routine ophthalmologic examination before the genetic confirmation of GSN amyloidosis.

The absence of family history or subtle eye problems may obscure the diagnosis of GSN amyloidosis in patients with lower cranial motor neuropathies or PBP. Notably, Caress et al. mentioned that hereditary gelsolin amyloidosis is easily omitted in the differential diagnosis of amyotrophic lateral sclerosis or bulbar MND.^4^ Furthermore, it has not been listed as a disease mimicking MND according to consensus guidelines and some research articles.^5^

Genetic testing is now widely available. This has allowed detection of de novo mutations in patients without a family history.^6^ In patient A, we could not establish an accurate diagnosis without genetic testing. Therefore, whole‐exome sequencing should be considered for patients with slowly PBP, even in the absence of family history, to prevent misdiagnosis.

## CONFLICT OF INTEREST

None of the authors have any conflicts of interest to disclose.

## ETHICS STATEMENT

We confirm that we have read the journal's position on issues involved in ethical publication and affirm that this report is consistent with those guidelines.

## Data Availability

Data openly available in a public repository that issues datasets with DOIs.
